# Gold removal from e-waste using high-intensity focused ultrasound

**DOI:** 10.1016/j.ultsonch.2024.107109

**Published:** 2024-10-16

**Authors:** Axi Holmström, Topi Pudas, Jere Hyvönen, Martin Weber, Kenichiro Mizohata, Tom Sillanpää, Joni Mäkinen, Antti Kuronen, Tapio Kotiaho, Edward Hæggström, Ari Salmi

**Affiliations:** a Electronics Research Laboratory, Faculty of Science, University of Helsinki, P.O.B. 64, FIN-00014 University of Helsinki, Finland; b Accelerator Laboratory, Faculty of Science, University of Helsinki, P.O.B. 43, FIN-00014 University of Helsinki, Finland; c Drug Research Program and Division of Pharmaceutical Chemistry and Technology, Faculty of Pharmacy, University of Helsinki, P.O.B. 56, FIN-00014 University of Helsinki, Finland; d Department of Chemistry, Faculty of Science, University of Helsinki, P.O.B 55, FIN-00014 University of Helsinki, Finland

**Keywords:** E-waste recycling, Urban mining, High-intensity focused ultrasound, Cavitation, Cavitation erosion, UN SDG 12

## Abstract

The demand for rare and precious metals (RPMs), e.g. gold, is increasing, as these are used in the ever-increasing amount of electronics needed for technological development and digitalization. Due to their rarity, virgin mining of RPMs is becoming more difficult and expensive. At the same time, over 62 Mt of e-waste is created globally each year. The high concentration of gold and other RPMs in e-waste makes it an excellent source for recycling. Unfortunately, current recycling methods need to separate the different metals and the current pyrometallurgical and hydrometallurgical processes also create toxic pollutants, large amounts of wastewater and require highly corrosive substances. Here we present a new method for gold removal for the purpose of recycling, using only water and high-intensity focused ultrasound to induce material erosion through cavitation. An 11.8 MHz ultrasonic transducer is used to first image the sample to locate gold-coated pads on discarded printed circuit boards (PCBs) and subsequently to remove only the gold layer. We demonstrate that the gold removal can be controlled by the number of transmitted ultrasonic bursts and that the energy efficiency is optimal when only minute amounts of the nickel layer beneath are also removed. Removing solely the gold layer also decreases the need for further processing steps. This greener gold removal method for e-waste is therefore well aligned with, and contributing to, the United Nations Sustainable Development Goal 12: Ensure sustainable consumption and production patterns.

## Highlights

• A new green method for gold removal from discarded PCBs (e-waste) is presented.
• High-intensity focused ultrasound (HIFU) removes gold from PCBs through cavitation.
• High-frequency HIFU (11.8 MHz) can remove only the gold from selected locations.
• Selective gold removal is achieved in water, without additional chemicals.

## Graphical abstract

**Figure:**
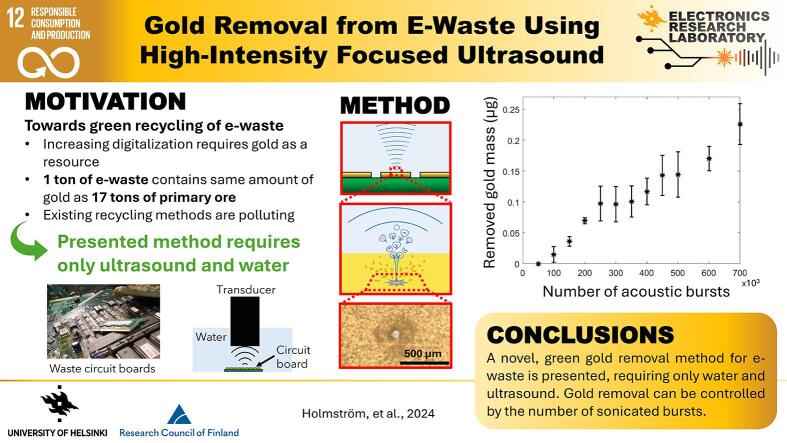

## 1 Introduction

Due to increasing technological development and digitalization, the demand for electronic devices is increasing. Electronics are built on printed circuit boards (PCBs), which contain rare and precious metals (RPMs), such as gold, silver, and platinum, as well as copper and nickel [1]. RPMs are not easily substituted for more available materials, as they have certain properties required for PCBs, such as high electrical conductivity and corrosion resistance. As the amount of electronics increases, so too does the amount of e-waste. Currently, over 62 Mt of e-waste is generated globally per year, of which only 14 Mt is recycled [2]. The amount of generated e-waste is expected to increase to 82 Mt per annum by 2030 [2]. E-waste recycling is already more profitable than virgin mining [3] and despite the low mass fraction of gold in e.g. PCBs (only 0.11 ‰), gold constitutes 66 % of the monetary worth [4].

In 2019, 4 831 t of gold was produced, with only 1 297 t coming from recycled sources [5]. With 55 000 t of unmined gold reserves and a current global annual mining rate of approx. 3 500 t, mineable gold reserves would run out in 15 years [5]. It is therefore necessary to increase gold recycling from e-waste. As gold (and other RPMs) is already concentrated in PCBs, they constitute an excellent source for recycling: There is approximately the same amount of gold in 1 t of e-waste as in 17 t of mineable ore [6]. Decreasing virgin mining and increasing recycling is therefore a key aspect of the United Nations Sustainable Development Goal (UN SDG) 12: Ensure sustainable consumption and production patterns, particularly Target 12.2: By 2030, achieve the sustainable management and efficient use of natural resources, and 12.5: By 2030, substantially reduce waste generation through prevention, reduction, recycling and reuse [7], [8].

Recycling e-waste is already being done, usually through pyrometallurgy and/or hydrometallurgy [1], [4], [9]. Such methods are needed, as waste PCBs contain several materials that need to be separated in different steps: plastic and other non-metallic substances are burnt off and all metals, e.g. copper, nickel, and gold, need to be separated. However, these methods have significant drawbacks: The burning of e-waste creates toxic and volatile air pollutants and large amounts of CO_2_ [1], [10], and hydrometallurgy usually employs toxic and/or highly corrosive substances for leaching (e.g. *aqua regia*, piranha solution (H_2_SO_4_/H_2_O_2_) and cyanide [10]), which are difficult to handle and create large amounts of wastewater [1], [10]. There are developments being made using biological substances, e.g. bioleaching [1], [11] and deep eutectic solvents [10], [12], that could provide greener solutions. However, these are still in the early development phase and are slow [11], [12]. The microorganisms are selective, i.e., one population only recovers one substance [11]. Furthermore, the microorganisms can perish due to the generated toxic substances, thus stopping the process [11]. Deep eutectic solvents again have high viscosity, which requires use at higher temperatures than inorganic acids and long leaching times [12], and some are not genuinely green, as their synthesis requires toxic components that are not easily biodegraded [12]. Thus, there is still a great need for improved and cleaner gold recycling methods.

In this paper, we present a novel high-intensity focused ultrasound (HIFU) technique to remove gold from PCBs, using only HIFU in water, requiring no additional chemicals. Breaking materials with HIFU-induced inertial cavitation has already been established in the medical fields of histo- and lithotripsy [13], [14], [15], [16]. Cavitation erosion on metals has also been studied [17], [18], [19], [20], [21], [22], [23], [24], [25], [26], but it has yet to become widely used for controlled material removal of metals. A recent study showed that cavitation activity could be utilized in a deep eutectic solvent to improve RPM recycling from e-waste [24]. However, the 20 kHz sonotrode in that study removed all metal layers of the PCB (containing gold, nickel, and copper) at once, requiring further separation steps. Watt *et al*. demonstrated removing gold nanoparticles and only minor amounts of nickel from a SIM card in a two-surfactant system, also by using a 20 kHz sonotrode [26]. While they managed to restrict the erosion depth to the layers of interest, the use of a sonotrode on a generic PCB sample is not sufficiently selective. While the surface of a SIM card consists almost exclusively of gold, generic gold pads on e.g. PCBs are much narrower than the tip of a sonotrode (tips in both [24], [26] were 6.4 mm). Thus, the adjacent substrate would also be removed, again requiring additional separation steps. In our approach, we use a high-frequency (11.8 MHz) HIFU transducer, which is first used to image a PCB sample to localize gold pads. Then the same transducer is driven at high power to cause cavitation-induced metal erosion from only desired areas, thereby removing the top gold layer (and only minute amounts of nickel from the layer beneath). Here we show how the gold and nickel removal depends on the number of transmitted ultrasonic bursts. This method requires only water (no added chemicals) and decreases the need for further processing steps by predominantly removing only gold. It could therefore contribute to greener and improved e-waste recycling methods and to the UN SDG 12. Table 1 summarizes some examples of research advancing HIFU-induced gold removal for recycling purposes, which have contributed towards the SDGs.

**Table 1:** Examples of some research that have advanced SDGs and HIFU-induced gold removal for recycling purposes.

**The focus of the study/studies**	**Key points**	**SDGs and targets**	**Countries of affiliation (authors)**	**Reference**
Histo- and lithotripsy using HIFU for biomedical applications	• Utilizing HIFU to non-invasively liquefy tissue or erode kidney stones	SDG-3: Ensure healthy lives and promote well-being for all at all ages. Target 3.8	Numerous groups world-wide	Review of various preclinical applications in both thermal and non-thermal regime [15].
Industry standard for conducting acoustic cavitation erosion tests	• Using 20 kHz sonotrode to induce cavitation erosion on e.g. metal (particularly Note 1 pertaining to “stationary specimen” tests) • Repeatable method to evaluate cavitation resistance in e.g. pumps, hydraulic turbines, valves, propellers etc.	SDG-9: Build resilient infrastructure, promote inclusive and sustainable industrialization and foster innovation. Target 9.1, 9.4	United States	[27], originally approved in 1972, latest ed. 2021.
Extracting gold nanoparticles from e-waste	• Reduce hazardous waste and energy requirements for gold nanoparticle synthesis by using a 20 kHz sonotrode • Extract the nanoparticles from e-waste (SIM card)	SDG-12: Ensure sustainable consumption and production patterns. Targets 12.2, 12.5	United States	[26]
Improved PCB recycling in deep eutectic solvent by low-power ultrasound	• Combining 20 kHz sonotrode with deep eutectic solvent for gold, nickel and copper recycling • Processing time for gold, nickel and copper removal improved thirtyfold	SDG-12: Ensure sustainable consumption and production patterns. Targets 12.2, 12.5	United Kingdom	[24]
Gold removal from e-waste using HIFU	• Proof-of-concept to demonstrate gold removal from PCBs using MHz HIFU • Ability to image and locate gold pads on a PCB and produce three deep erosion holes • Done in water immersion without added chemicals	SDG-12: Ensure sustainable consumption and production patterns. Targets 12.2, 12.5	Finland	[28] (conference paper), precursor to the study presented in this paper.

## 2 Methods

### 2.1 Setup and samples

**Fig. 1:**
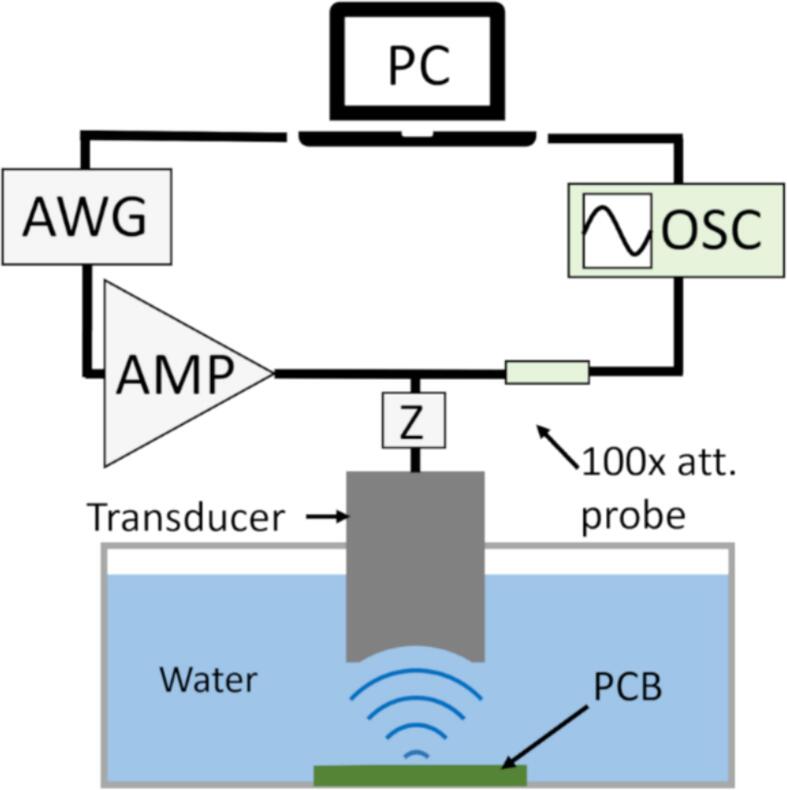
Setup. A PC controlled a three-axis translation stage and the arbitrary waveform generator (AWG), which transmitted excitation signals to the 11.8 MHz custom-built focused transducer. A power amplifier (AMP) was used for erosion experiments and impedance matching (Z) ensured maximum power transfer. In imaging and when positioning the transducer, the echoes reflected from the printed circuit board (PCB) were received with an oscilloscope (OSC) and recorded on the PC. A 100x attenuating probe was used to protect the oscilloscope from the transmitted high-amplitude (*U*_PP_ = 398 V) signals in the erosion experiments.

A schematic of the setup used in this work is shown in Fig. 1. The setup is an improved version of the ones described in [28], [29], [30], [31] (gold removal proof-of-concept conference paper [28], aluminium erosion [30], [31], soft material removal [29]). The same transducer was used for both imaging (locating gold pads) and material erosion. It was custom-built using a commercial curved piezo bowl (type Pz26, model F5260195, CTS Ferroperm, Denmark), with epoxy backing in a 3D printed housing. The transducer had a central frequency of 11.8 MHz, bandwidth of 1.2 MHz, element diameter 1.9 cm, focal distance 1.5 cm and focal width 140 μm. All sonications were performed in an open container with purified water (RiOs Essential Water Purification Systems, Merck KGaA, Germany) that had been vacuumed (Laboport UN 810.3 FTP, KNF Neuberger GmbH, Germany) for 30 min prior to the start of experiments. This was done to obtain similar conditions for bubble nucleation in all cavitation experiments, as both particulates and dissolved gas content influence the cavitation probability.

A motorized three-axis translation stage (NLS4 NEMA 17 MDrive, Newmark Systems Inc., USA) was used to move the transducer. Transmission, signal acquisition, and the translation stage were all controlled by a custom-written Python script (Python 3). On the transmission side, an arbitrary waveform generator (AFG31052 SERIES, Tektronix, USA) was used to generate the desired signals, which were amplified with a Class A RF power amplifier (500A100A, Amplifier Research, USA). During imaging, minimum amplification was used (0 % RF gain), whereas during erosion experiments, maximum settings were used (100 % RF gain). At maximum amplification, the peak-to-peak voltage of the electric signal driving the transducer was *U*_PP_ = 398 V (example signal shown in Supplementary material Fig. S1a). The transducer was matched to 50 Ω (at the driving frequency) with an LC impedance matching circuit to ensure maximum power transfer from the 50 Ω output of the amplifier to the transducer. During imaging, the echoes reflected from the sample were recorded with a digital oscilloscope (Picoscope 5442D, Pico Technology, UK) and saved on the PC. A 100x attenuating probe (TT-HV250, TESTEC Elektronik GmbH, Germany) was used to protect the oscilloscope from the transmitted high-amplitude signals in the erosion experiments. Echoes were also displayed in real time during erosion experiments for cavitation monitoring.

To determine the acoustic pressure at the focus, an optical hydrophone (ONDA HFO-690, Ø = 100 μm, Onda Corporation, USA) was used to measure the pressure directly at the focus of the transducer. Due to the high pressure amplitudes involved, cavitation started after only three rarefactive cycles (four compressive), which was repeatable over all 15 recorded signals (see Supplementary materials S1 and Fig. S1b for details). However, at the third peaks (both positive and negative), the electric signal was almost equal to the stable oscillation values and thus the peak-positive and peak-negative-pressures, *P*_PPP_ and *P*_PNP_, could be estimated. When the pressure values were calculated, the Small Signal Sensitivity *SSS* = 5.57 mV/MPa provided by the device was used with a frequency-dependent tip scattering correction, *k* = 0.459, calculated for 11.8 MHz. The corrected pressure was calculated as *P*_corrected_ = *k*·*P*_SSS_, where *P*_SSS_ was the pressure obtained using only the *SSS* (for details, see Supplementary materials S1). Thus, the peak-positive and peak-negative pressures (marked with green asterisks in Fig. S1b) were obtained: *P*_PPP_ = (46 ± 1) MPa and *P*_PNP_ = (−35 ± 5) MPa (mean ± 1 SD), with standard deviations calculated from the 15 signals.

**Fig. 2:**
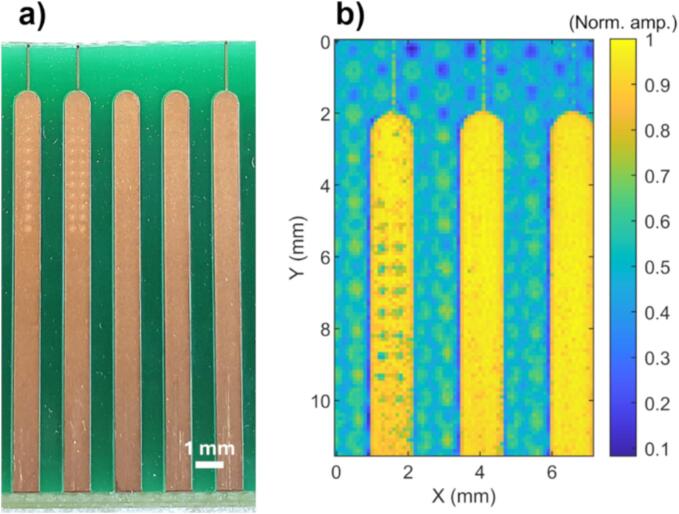
Locating gold pads. a) Example photograph of the PCB and b) image taken with the 11.8 MHz transducer. Erosion holes from prior experiments can also be seen in both images.

The PCB sample (Fig. 2a), containing several gold pads, was cut from a discarded motherboard. The gold layer thickness of the pads was measured with Rutherford backscattering spectrometry using a 3 MeV proton beam from 5 MeV tandem accelerator at the University of Helsinki (incident angle α = 30°). Scattered probing particles were detected using an Ortec Ultra ion-implanted silicon detector (scattering angle θ = 165°, detector-sample distance 80 mm, 50 mm^2^ detector surface area, limiting detector solid angle to 8 msr). The gold layer thickness was measured to be (1.73 ± 0.04) µm.

### 2.2 Identifying regions-of-interest

The PCB was imaged prior to each erosion experiment to determine the locations of the gold pads. Imaging was performed with the same 11.8 MHz transducer that was used for erosion. Imaging parameters were *f* = 11.8 MHz, 10 cycles per burst, step size 100 µm. An example photograph and an ultrasonic image of the sample are shown in Fig. 2a and b, respectively. The gold pads are clearly visible and distinguishable from the adjacent substrate. In addition, erosion holes from prior experiments can also be seen in both images. Even with the 11.8 MHz transducer, all but the smallest erosion holes are discernible and pristine pads thus easily identified.

### 2.3 Gold removal

#### 2.3.1 Sonication parameters

Two sets of experiments were performed relating to gold removal: 1) Defocus sweep to determine the optimal transducer-sample distance, and 2) a burst sweep to evaluate gold removal. Preliminary experiments revealed that material erosion was dependent on the transducer-sample distance, hence the defocus sweep was performed. In the defocus sweep, the constant sonication parameters, used at each sonication spot, were: *f* = 11.8 MHz, maximum amplitude (*U*_PP_ = 398 V, *P*_PPP_ = 46 MPa, *P*_PNP_ =  − 35 MPa), 50 cycles per burst, 500 000 bursts per sonication spot, pulse repetition frequency (PRF) 500 Hz. Three repetitions were made with 13 defocus distances in 100 µm steps, from defocus − 500 µm (focus inside the sample) to +700 µm (focus on top of the sample), with defocus 0 µm signifying focusing on the sample surface.

Having determined the optimal defocus distance (+150 µm, see 3. Results), the burst sweep was performed. The purpose was to determine how gold removal depends on input energy. Only the number of bursts sonicated in one spot was varied, the other sonication parameters were constant: *f* = 11.8 MHz, maximum amplitude (*U*_PP_ = 398 V, *P*_PPP_ = 46 MPa, *P*_PNP_ =  − 35 MPa), 50 cycles per burst, PRF 500 Hz, defocus distance +150 µm. Three repetitions were performed with (50, 100, 150, 200, 250, 300, 350, 400, 450, 500, 600, 700) × 1000 bursts per spot. Cavitation activity, which was monitored from the echoes, did not cease during sonications.

#### 2.3.2 Quantifying erosion areas, volumes, and mass

**Fig. 3:**
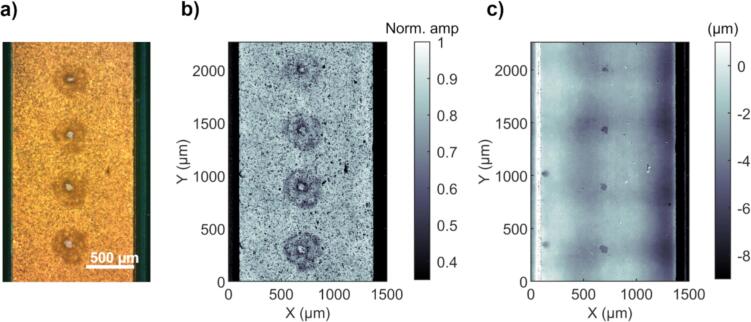
Examples of a) optical microscope image, b) CESAM amplitude map, and c) CESAM topography map of four corresponding holes from one burst sweep. The surface roughening around the deep holes is visible in both the optical image and amplitude map (a, b). The small deep holes, where gold has been removed and the nickel beneath is exposed, are readily visible in the optical image (a) and the topography map (c). Erosion areas and volumes were subsequently determined from the topography maps.

All erosion holes were measured with a coded-excitation scanning acoustic microscope (CESAM) [28], [32], [33]. Produced topography maps enabled calculating both the area of the erosion holes (which were also visible in optical images, Fig. 3a), and their depth, and hence volume, as well. The CESAM images were obtained with a 375 MHz transducer (bandwidth 140 MHz, beam width 2.5 μm, depth-of-focus 29 μm, scanning step size 2 μm) using a 300–500 MHz linear frequency modulated chirp of 1 µs length with a Gaussian envelope. Examples of an optical image and corresponding amplitude and topography maps taken with CESAM are shown in Fig. 3. The surface roughening is seen in both the optical and CESAM amplitude images, whereas the deep erosion holes, where the nickel beneath the gold pad is exposed in the optical image, are readily visible in the CESAM topography map. Thus, the CESAM topography maps were used to analyze eroded areas and volumes of the deep erosion holes.

All analyses were done with Matlab R2020b (The MathWorks, Inc., USA). As the gold pads are not entirely flat (up to 5 µm surface height variation within one pad, an example pad is shown in Supplementary material Fig. S2), each erosion hole was first processed manually to determine the correct zero level of the surface. Next, the areas of the deep erosion holes (visible in the topography map) were determined by manually creating a mask of the hole using Matlab’s “drawassisted” function. The area was then calculated from the mask and the erosion hole volume was calculated from the topography inside the mask. The volume of removed gold was subsequently calculated from the erosion hole topography using the measured gold layer depth *d*_Au_ = (1.73 ± 0.04) µm to determine when gold had been removed and when the erosion had reached the nickel beneath. The mass of removed gold was then calculated from the eroded gold volume using the density of gold, ρ_Au_ = 19.32 g/cm^3^ [34]. Gold removal efficiency was also calculated as the ratio of removed gold mass to used electrical energy (and normalized to the maximum mean value). The electrical energy delivered to the transducer was 1.7 mJ per burst, as determined from the measured voltage signals (for details, see Supplementary materials S3).

## 3 Results

### 3.1 Transducer-sample distance

**Fig. 4:**
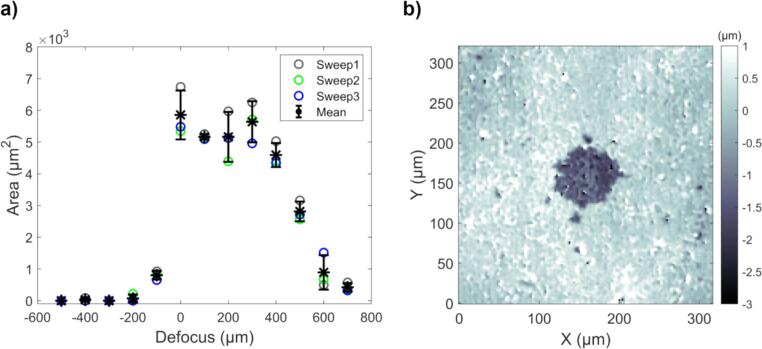
Defocus sweep to determine optimal transducer-sample distance. a) Erosion area as a function of defocus distance. Sweep numbers signify measurement series and the mean ± 1 SD is shown. Defocus 0 µm is focused at the surface, negative defocus below and positive defocus above the surface. The largest eroded areas are achieved with defocus between 0 and +300 µm. b) Example of topography map of a hole at +100 µm defocus. The width of the hole is slightly less than the 140 µm focal beam width. Only a few stochastic cavitation events (smaller pits) are visible outside the main lobe.

First, the optimal transducer-sample distance for gold erosion was determined from the defocus sweep. The results from all three sweeps and their mean ± 1 SD are shown in Fig. 4a. The largest eroded areas were achieved with between 0 µm and +300 µm defocus (0 µm signifying focusing on the surface, positive moving the focus above the surface). In this region, the widths of the erosion holes approach the width of the focal main lobe (140 µm), and only few stochastically generated separate minor pits outside the large erosion areas are present. A CESAM image of one hole produced with +100 µm defocus is shown as an example in Fig. 4b. Based on these results, a defocus of +150 µm was used in subsequent erosion experiments.

### 3.2 Gold removal

**Fig. 5:**
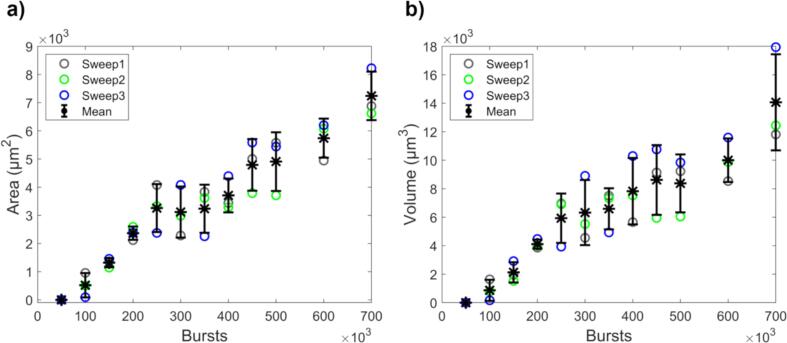
Material removal as a function of number of sonicated bursts. a) Area and b) volume of deep erosion holes. Sweep numbers signify measurement series and the mean ± 1 SD is shown.

Having determined the optimal defocus distance (selected as +150 µm), the main study, i.e. gold removal as a function of sonicated bursts, was performed. As expected, the erosion area increased with number of bursts, as more bursts enables more cavitation events and thus erosion (Fig. 5a). The total eroded volume (total volume of each erosion hole, including gold and nickel) was calculated using the depth information obtained with the CESAM (Fig. 5b).

**Fig. 6:**
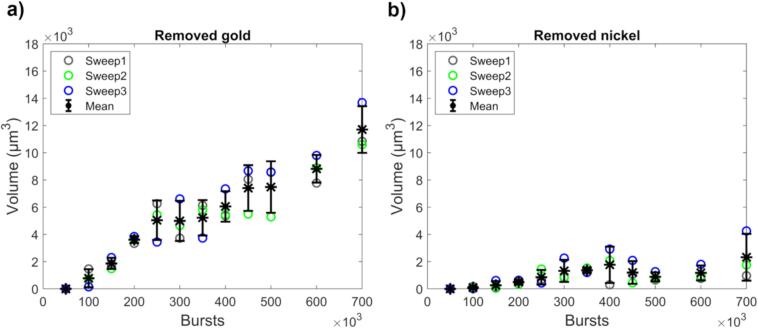
Volumes of removed a) gold and b) nickel as a function of number of bursts. Sweep numbers signify measurement series and the mean ± 1 SD is shown. Gold removal plateaus slightly as the erosion continues into the nickel layer beneath. Gold removal still continues, as the weaker cavitation activity in the outer region of the focus continues to remove gold.

The volume of removed gold was subsequently calculated (Fig. 6a). With an increasing number of bursts, more gold was removed, though a slight plateauing effect is seen. This effect occurs because the region of highest cavitation activity (highest pressures in the focus) erodes through the (1.73 ± 0.04) µm gold layer and starts eroding the nickel beneath. The gold erosion does not halt completely, as the weaker cavitation activity in the outer region of the focus continues to remove gold. For gold recycling purposes, it would be desirable to only remove the gold, without removing nickel. Nickel starts to be removed at approx. 250 000 bursts (Fig. 6b). Thus, sonicating fewer bursts in one spot and then moving the transducer could be preferable to sonicating many bursts in one spot. One can also conclude that while cavitation is a stochastic phenomenon, at these high burst counts, the gold removal is quite repeatable.

**Fig. 7:**
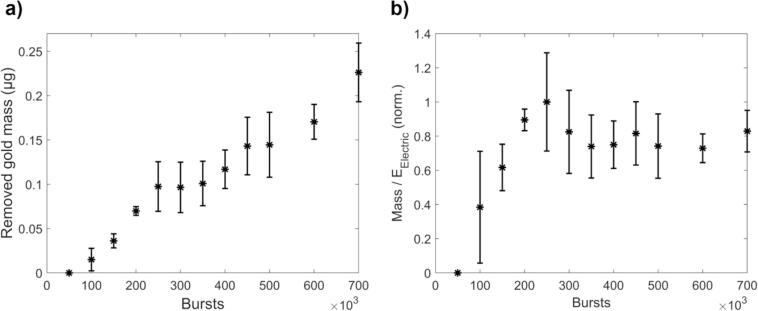
Mass of removed gold and removal efficiency as a function of bursts. Mean ± 1 SD from the three sweeps in Fig. 5, Fig. 6a are shown. **a)** Mass of removed gold. **b)** Removal efficiency: The ratio of removed gold mass to used electrical energy. For readability, the values have been normalized by the maximum mean value (at 250 000 bursts). Beyond 250 000 bursts, the efficiency decreases as nickel is also removed.

Finally, the mass of the removed gold was calculated (Fig. 7a). Despite the small masses removed from each hole, erosion was achieved through the depth of the gold layer. The removed gold mass was also compared to the electrical energy used (Fig. 7b), which showed that the removal efficiency starts to decrease after 250 000 bursts, when nickel is also removed. Therefore, even though gold continues to be removed with more bursts, but at a slower rate (outside the main focal volume), the removal efficiency decreases as more electric energy is wasted on removing nickel instead of gold inside the main focal volume.

## 4 Discussion

As technological development and digitalization continues on a global scale, the need for PCBs, and subsequently RPMs (e.g. gold), will continue to increase. As current e-waste recycling methods still have significant drawbacks (creation of toxins, wastewater, use of strong acids, etc. [1], [10]), new, more environmentally and user-friendly recycling methods could greatly contribute to the UN SDG 12 (Ensure sustainable consumption and production patterns), particularly Targets 12.2 and 12.5 [7], [8]. The HIFU method presented in this paper — using only ultrasound in water, with no added chemicals — could be a step towards such a solution.

### 4.1 Benefits of HIFU

Whereas HIFU-induced cavitation is common in medicine, applications for metal erosion are scarce. While cavitation damage to metals has been long known [35], [36], previous work on controlled cavitation-induced metal erosion has mainly focused on studying laser-generated single-bubble erosion dynamics [18], [20], [22], [25], [37], [38] or the effect of non-localized ultrasound [26] and adding it to existing methods [24]. Here we showed that high-frequency HIFU (11.8 MHz) can be used to locally erode gold and that the amount of removed material can be controlled by the number of sonicated bursts. Our previous proof-of-concept work on HIFU gold removal demonstrated three erosion holes created with identical acoustic parameters, without studying the controllability of the erosion [28]. Here we showed the gold erosion’s dependence on easily controlled parameters, i.e., defocus and number of bursts. Our tightly focused transducer (focal width 140 µm) also enabled ultrasonic imaging to locate the gold pads prior to erosion, thus facilitating gold removal from only the desired area, i.e. the gold pads, and not the surrounding substrate. While sonotrodes have many advantages (e.g. that they are built for high amplitude and long sonication times), they lack the selectivity of HIFU. This selectivity is an important aspect, because the need for pyrometallurgical and hydrometallurgical techniques in current e-waste recycling processes stems from the presence of undesired materials (e.g. plastics) and other metals present in PCBs (e.g. copper and nickel). Removing only gold (and possibly minute amounts of nickel) therefore decreases the need for hazardous post processing.

### 4.2 Gold removal and cavitation

The presented burst sweep results show that the amount of removed gold initially increases linearly, but shows a slight plateauing effect when nickel starts to be removed. This was expected, as more bursts translates to more cavitation events. Gold removal did not stop completely, even when nickel was also removed. The cavitation probability is not constant in the entire focus, but pressure dependent, and hence the number of cavitation events decreases towards the outer edges of the focus, even within the main lobe. This outer region continued to erode gold with the highest burst counts. Bubble cloud dynamics are the cause of the optimal defocus distance – when focusing some distance above the surface, the created bubble cloud with its subsequent internal dynamics caused by cavitation events (shock waves and microjets) create suitable conditions for gold erosion. While the effect of the stand-off distance of single bubbles on erosion has been studied [18], [20], [22], [25], [37], our case is not that of a single bubble, but a cloud, and hence such results are not directly transferrable. In this work, we empirically determined a repeatable optimal defocus region for use in our gold removal application. High-resolution high-speed camera footage and analyses similar to [39] could provide insight into the bubble cloud dynamics and why the optimal defocus is between 0 µm and +300 µm.

Only the effect of defocus and burst count was investigated in this study, even though other parameters affect cavitation erosion too: frequency, pressure amplitude, number of cycles, PRF, dissolved gas in the liquid, etc. We used a high-frequency (11.8 MHz) focused transducer to ensure small spot size (much smaller than the gold pad, to ensure selectivity) and high energy density at the focus, even though the cavitation threshold increases with frequency [40], [41], [42]. Higher amplitudes might decrease the number of required bursts, as each burst could generate more powerful microjets and inertial bubble collapses. An amplitude study was excluded since even using the highest driving voltages generated by our commercial amplifier, several hundred thousand bursts were still required. The number of cycles also affects the erosion, as long bursts might cause bubble shielding [43], which could reduce erosion. As we could not monitor the cavitation cloud dynamics during the bursts, the effect of the burst length in our case remains undetermined. The transducer took ∼ 20 cycles to settle into stable oscillation, so 50 cycles/burst was selected, which also demonstrated effective gold removal in preliminary experiments. The water was always degassed for 30 min prior to sonications, but as the sonication experiments took 2–4 h to perform, the gas content did not remain constant during the experiments. This could explain some variability in the results. The main reason for the long sonication times was the low PRF (500 Hz), which was chosen to ensure sufficient heat dissipation from the transducer. Higher PRFs with these high amplitudes caused our thin curved piezos to overheat and break.

Inertial cavitation is affected by many parameters, which gives it a quasi-stochastic nature. Thus, the variability between repeated experiments introduces the greatest uncertainty in the results. By manually performing the erosion area selection, uncertainties in area were at the level of some *per mille*. Even the uncertainty in the gold-layer depth measurement only caused 1–2 % uncertainty in the volume estimates. Thus, these are minute compared to the variability shown between repeated measurements.

Compared to our previous studies on cavitation erosion on aluminium [30], [31], gold is surprisingly difficult to erode. Aluminium can be eroded using only a few percent of the number of bursts required for gold (setup in [30], [31] was comparable to [28]). One hypothesis is that since gold is more malleable (less brittle) than aluminium, the erosion therefore becomes a fatigue process [44]. The high number of required bursts could support this. It is also possible that the layered structure of the PCB pads plays a role in the fracture mechanics of the top layer (gold). As gold is a noble metal, it might also be resistant to any contributing effect from free radicals that form in the water during inertial cavitation [45]. Molecular dynamics simulations could provide valuable insight into the cavitation erosion dynamics of different solid materials. In the presented method, gold erosion could be accelerated by introducing abrasive particles to the water. Other methods for improving the erosion could be to decrease frequency while maintaining a sufficiently tight focus (to reduce the frequency-dependent cavitation threshold, but reaching high energy densities) and to use larger piezo disks to generate higher power.

### 4.3 Outlook and scale-up

In this study, we focused on the HIFU gold erosion and excluded the subsequent gold collection. While developing the gold collection is important for any future use of this technology, the novelty lies in using HIFU for selective gold removal. Collecting the removed gold was left for future work. There are several common methods for collecting small particulates from aqueous solutions, e.g. filtering, sedimentation, evaporation, and centrifugation. More complex methods for gold particle collection also exist, e.g. nylon-based 3D-printed scavengers [46], amyloid nanofibril aerogels [47], and metal–organic frameworks (e.g. [48]). Localized sampling during the sonication process [29] could also be combined with any of the aforementioned methods to reduce the volume from which to collect the gold. As gold was not collected, the purity and size of the removed gold particles is not known, which will also be studied in the future. If the gold would be removed as nanoparticles, as in [26], it would open up even more possible new end-uses for the removed gold.

This method’s greatest advantage is the use of only ultrasound and water, with no added chemicals, and that it allows to remove gold from accurately predefined areas of e.g. a PCB. Despite the amounts of removed gold now being small, the technology could be optimized and scaled up by using several transducers in parallel. Optimization could be done by e.g. tuning the used frequency (lower frequencies have lower cavitation thresholds) and the focusing of the used transducer. A lower-frequency transducer with the same focusing (numerical aperture) has a larger focal width. Thus, it could plausibly remove gold from a larger area in the same amount of time. Considering the added beneficial effect of a lower cavitation threshold, gold removal might be increased further. When decreasing frequency, care must be taken to ensure both sufficient energy densities at the focus and a small enough spot size to maintain selectivity. This could be done with a more tightly focused transducer. After appropriate single-transducer optimization, many transducers could be used simultaneously to scale up the technology. These could be placed in parallel (in suitable places for sonicating different PCBs simultaneously) or combined into an array to achieve much higher acoustic intensities.

A rough estimate of the profitability, i.e., the monetary worth of the removed mass of gold divided by the cost of electricity used, was > 1 for all cases where any gold was removed, even when considering a 40–60 % efficiency of the power amplifier. With further optimization and scaling, the presented environmentally friendly gold removal method could therefore become economically viable and contribute to improving e-waste recycling. Furthermore, this technology could be used to remove other metals as well in a layer-by-layer sequence, e.g., first removing gold, then nickel, then copper and so forth. A sequential approach is already necessary for pyrometallurgy, bioleaching, and deep eutectic solvents to separate the different metals in e-waste. Hence, our method could also be added to other processes. It thus aligns well with UN SDG 12: Ensure sustainable consumption and production patterns.

## 5 Conclusion

A novel, green gold removal method for e-waste, based on HIFU-induced cavitation, was presented. Its benefits arise from using no chemicals (only water) and selectively removing only gold (and only minute amounts of nickel) from gold pads on discarded PCBs. The selectivity is achieved by first imaging the sample with the HIFU transducer to locate the gold pads and subsequently removing only the gold layer – only from the pads. The gold removal can be controlled by the number of sonicated bursts. With the increasing accumulation of e-waste and demand for PCBs, improved gold recycling methods are needed. Thus, this method could contribute to the United Nations Sustainable Development Goal 12: Ensure sustainable consumption and production patterns.

## CRediT authorship contribution statement

**Axi Holmström:** Writing – original draft, Visualization, Supervision, Methodology, Investigation, Funding acquisition, Formal analysis, Conceptualization. **Topi Pudas:** Writing – review & editing, Software, Methodology, Investigation. **Jere Hyvönen:** Writing – review & editing, Visualization, Software, Methodology, Investigation. **Martin Weber:** Writing – review & editing, Methodology, Investigation. **Kenichiro Mizohata:** Writing – review & editing, Methodology, Investigation. **Tom Sillanpää:** Writing – review & editing. **Joni Mäkinen:** Writing – review & editing, Formal analysis. **Antti Kuronen:** Writing – review & editing, Supervision, Conceptualization. **Tapio Kotiaho:** Writing – review & editing, Supervision, Conceptualization. **Edward Hæggström:** Writing – review & editing, Supervision. **Ari Salmi:** Writing – review & editing, Supervision, Funding acquisition, Conceptualization.

## Declaration of competing interest

The authors declare the following financial interests/personal relationships which may be considered as potential competing interests: Ari Salmi, Axi Holmström, Joni Mäkinen, Jere Hyvönen, Tom Sillanpää, Tapio Kotiaho, Antti Kuronen, Edward Hæggström has patent #WO2023281159A1 pending to University of Helsinki. Ari Salmi, Jere Hyvönen, Joni Mäkinen, Antti Kuronen, Axi Holmström, Topi Pudas, Tom Sillanpää, Tapio Kotiaho, Edward Hæggström has patent #WO2024062155A1 pending to University of Helsinki. If there are other authors, they declare that they have no known competing financial interests or personal relationships that could have appeared to influence the work reported in this paper.

## Data Availability

Data will be made available on request.
