# Supplementary material for: Gold removal from e-waste using high-intensity focused ultrasound
Source: Ultrason Sonochem. 2024 Oct 16;111:107109. doi: 10.1016/j.ultsonch.2024.107109 (PMC11532776; doi:10.1016/j.ultsonch.2024.107109)
Supplement: Supplementary Data 1 [file mmc1.docx]

Appendix A. Supplementary materials

**Gold Removal from E-Waste Using High-Intensity Focused Ultrasound**

Axi Holmström^a^*, Topi Pudas^a^, Jere Hyvönen^a^, Martin Weber^a^, Kenichiro Mizohata^b^, Tom Sillanpää^a,c^, Joni Mäkinen^a^, Antti Kuronen^b^, Tapio Kotiaho^c,d^, Edward Hæggström^a^, Ari Salmi^a^

^a^ Electronics Research Laboratory, Faculty of Science, University of Helsinki, P.O.B. 64, FIN-00014 University of Helsinki, Finland.

^b^ Accelerator Laboratory, Faculty of Science, University of Helsinki, P.O.B. 43, FIN-00014 University of Helsinki, Finland.

^c^ Drug Research Program and Division of Pharmaceutical Chemistry and Technology, Faculty of Pharmacy, University of Helsinki, P.O.B. 56, FIN-00014 University of Helsinki, Finland.

^d^ Department of Chemistry, Faculty of Science, University of Helsinki, P.O.B 55, FIN-00014 University of Helsinki, Finland.

*Corresponding author: [axi.holmstrom@helsinki.fi](mailto:axi.holmstrom@helsinki.fi), Electronics Research Laboratory, Faculty of Science, P.O.B. 64, FIN-00014 University of Helsinki, Finland

**S1: Voltage and pressure measurement with maximum amplitudes at the focus**

The voltage and pressure measurements were conducted by driving the transducer (impedance matching included) with maximum settings in water. The maximum input voltage and maximum RF gain (set to 100 %) of the power amplifier (500A100A, Amplifier Research, USA) was used. The voltage signals were stable, and four signals were recorded. An example signal is shown in Fig. S1a. The transducer reaches a maximum amplitude after 5 cycles and settles after 2 µs (20 cycles). To determine the stable peak-to-peak amplitude *U*_PP_, one positive peak and its subsequent negative peak were selected manually at ~ 3 µs for each signal. From these values, the peak-to-peak voltage was calculated to be *U*_PP_ = (397.6 ± 0.4) V. As the driving electronics can drift during long sonications, the voltage was reported as an integer value of *U*_PP_ = 398 V.

The maximum pressure was measured directly at the focus using an optical hydrophone (ONDA HFO-690, Ø = 100 μm, Onda Corporation, USA) and 15 signals were recorded (Fig. S1b). As is seen in Fig. S1b, cavitation commenced after 3-4 cycles (3 rarefactive, 4 compressive), after which the signal was lost. At the third positive and negative peaks, the hydrophone signal was still reasonably stable and the voltage at these peaks were also close to the stable voltage values (10-15 % difference). The peak-positive and peak-negative-pressures, *P*_PPP_ and *P*_PNP_, were estimated from these peaks (marked with green asterisks in Fig. S1b). To convert the hydrophone’s voltage signals to pressure, the Small Signal Sensitivity *SSS* = 5.57 mV/MPa provided by the device and a frequency-dependent tip scattering correction were used (T = 20.3°C) [1]. As the signals end abruptly due to onset of cavitation, the signals were not processed entirely in the frequency domain as described in [1], which introduces errors without careful windowing. Instead, the pressure was first calculated from the voltage signal using the *SSS* to obtain *P*_SSS_, and then corrected with the frequency-dependent tip scattering correction factor *k* = 0.459 calculated for 11.8 MHz, i.e. *P*_corrected_ = *k*·*P*_SSS_. Thus, the peak-positive and peak-negative pressures (marked with green asterisks in Fig. S1b) were obtained: *P*_PPP_ = (46 ± 1) MPa and *P*_PNP_ = (−35 ± 5) MPa (mean ± 1 SD).


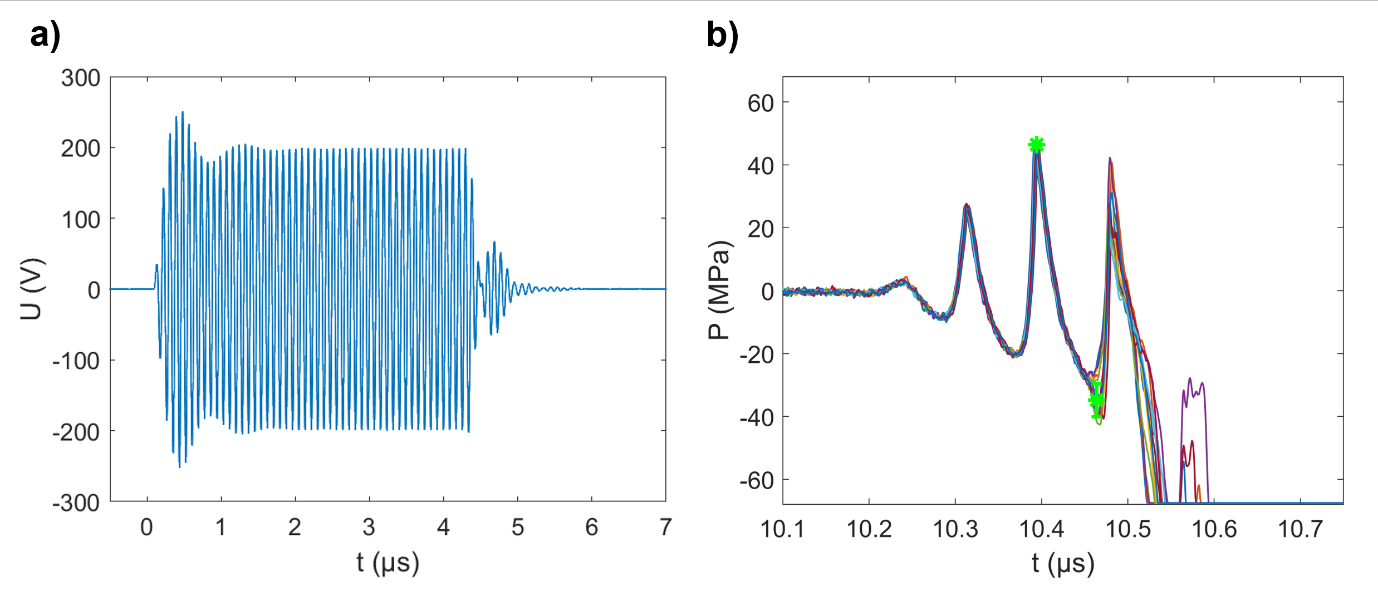


Fig. S1. Maximum voltage and pressure when measured at maximum amplitude at the focus. **a)** Example voltage signal. The *U*_PP_ = 398 V was determined from consecutive positive and negative peaks at ~ 3 µs from four measured signals. **b)** 15 pressure signals measured at the focus. The signal was lost when cavitation began (after 10.5 µs). The peaks used to estimate *P*_PPP_ and *P*_PNP_ are indicated with green asterisks, giving *P*_PPP_ = (46 ± 1) MPa and *P*_PNP_ = (−35 ± 5) MPa (mean ± 1 SD of the signals). The hydrophone signal begins at 10.2 µs, which is the travel time of the sound to the focus 15 mm from the transducer.

**S2: Example of CESAM images from one sonicated gold pad**

**
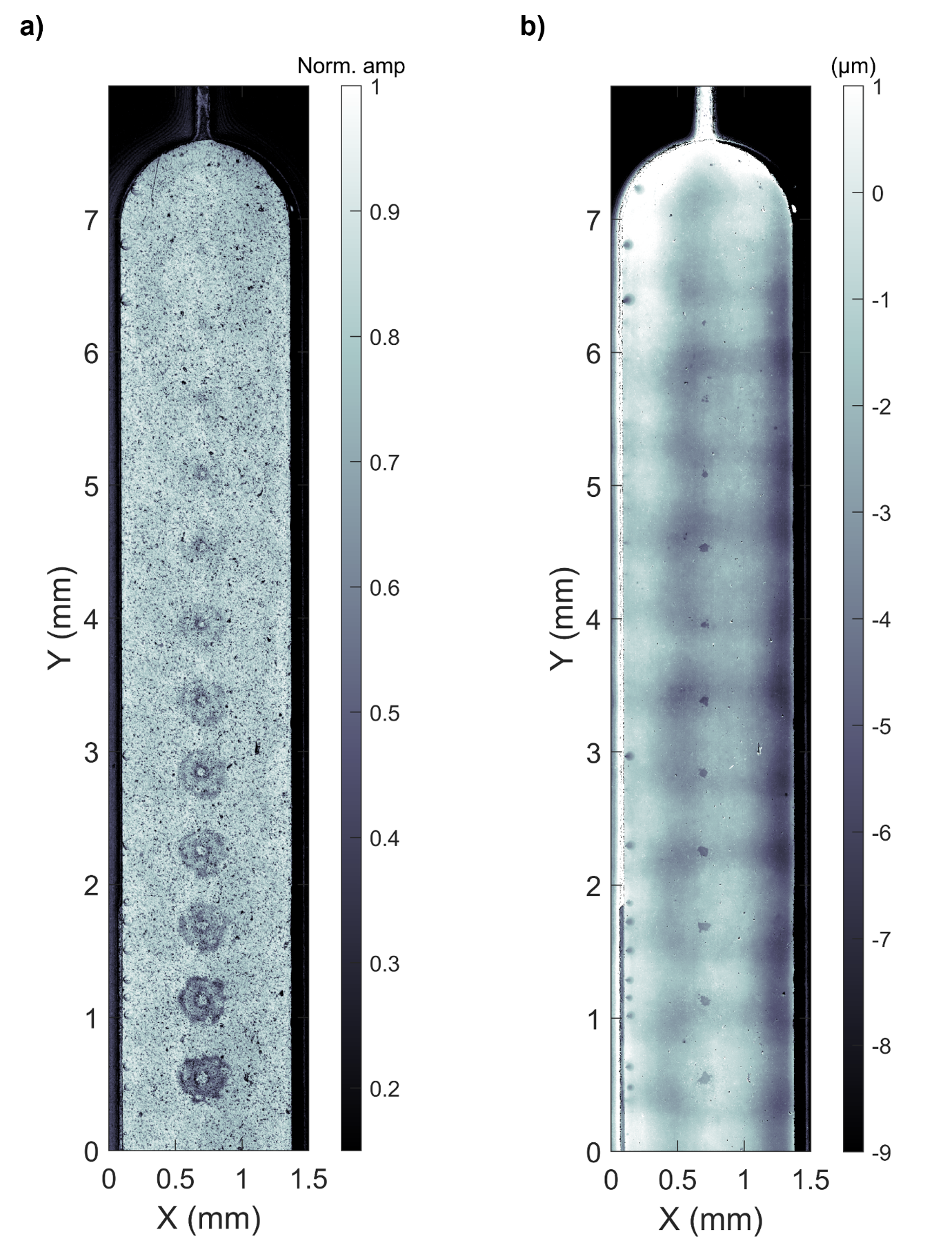
**

Fig. S2. CESAM images of one gold pad from one burst sweep. The number of bursts increases from top to bottom. Both the **a)** amplitude map and the **b)** topography map are obtained simultaneously. The surface roughening is clearly seen in the amplitude map and the deep holes at the center of the erosion mark, used for removal analysis, are also visible in the topography map. Fig. 3b & c are cut from the region between Y = 1.9 mm and Y = 4.2 mm. The erosion hole shown in Fig. 4b is from a different gold pad, but imaged with CESAM with the same parameters as shown here. The resolution is thus identical in all CESAM images.

**S3: Calculation of used electrical energy**

To determine the electrical energy used at the transducer, the four voltage signals (one example shown in Fig. S1a) were used. As the signals were not perfect sinusoids, the energy was calculated for each as the integral

$$E_{elec}=\int_{t_{1}}^{t_{2}} \frac{{u(t)}^{2}}{R}dt$$

where *u(t)* is the voltage signal, *t*_1_ and *t*_2_ are the beginning and end times of the signal, respectively (where the signal first exceeds and last returns to 0 V), and *R* is the resistance of the load (transducer). Because of the impedance matching to the transducer, a purely resistive load of *R* = 50 Ω was used. This might be slightly inaccurate, but should not introduce errors larger than a few percent. The obtained electrical energy was 1.7 mJ per burst (the difference in energies between signals was < 2 ‰).

To calculate the energy efficiency of the gold removal for different bursts, the masses (mean ± 1 SD) of removed gold were divided by the electrical energy used (i.e., multiplying the number of bursts with 1.7 mJ per burst) and normalized to the maximum mean value.

**References**

[1] ONDA HFO-690 User’s manual, section 2. Principle of operations, eq. 1-12.
